# Monitoring of Xenograft Tumor Growth and Response to Chemotherapy by Non-Invasive In Vivo Multispectral Fluorescence Imaging

**DOI:** 10.1371/journal.pone.0047927

**Published:** 2012-10-24

**Authors:** Henrike Caysa, Stefan Hoffmann, Jana Luetzkendorf, Lutz Peter Mueller, Susanne Unverzagt, Karsten Mäder, Thomas Mueller

**Affiliations:** 1 Department of Internal Medicine IV (Oncology/Hematology), Martin-Luther-University Halle-Wittenberg, Halle/Saale, Germany; 2 Institute of Pharmacy, Martin-Luther-University Halle-Wittenberg, Halle/Saale, Germany; 3 Institute of Medical Epidemiology, Biostatistics and Informatics, Martin-Luther-University Halle-Wittenberg, Halle/Saale, Germany; Univesity of Texas Southwestern Medical Center at Dallas, United States of America

## Abstract

A continuous monitoring of the whole tumor burden of individuals in orthotopic tumor models is a desirable aim and requires non-invasive imaging methods. Here we investigated whether quantification of a xenograft tumor intrinsic fluorescence signal can be used to evaluate tumor growth and response to chemotherapy. Stably fluorescence protein (FP) expressing cell clones of colorectal carcinoma and germ cell tumor lines were generated by lentiviral transduction using the FPs eGFP, dsRed2, TurboFP635, and mPlum. Applying subcutaneous tumor models in different experimental designs, specific correlations between measured total fluorescence intensity (FI) and the tumor volume (V) could be established. The accuracy of correlation of FI and V varied depending on the cell model used. The application of deep-red FP expressing xenografts (TurboFP635, mPlum) was observed to result in improved correlations. This was also reflected by the results of a performed error analysis. In a model of visceral growing mPlum tumors, measurements of FI could be used to follow growth and response to chemotherapy. However, in some cases final necropsy revealed the existence of additional, deeper located tumors that had not been detected *in vivo* by their mPlum signal. Consistently, only the weights of the tumors that were detected *in vivo* based on their mPlum signal correlated with FI. In conclusion, as long as tumors are visualized by their fluorescence signal the FI can be used to evaluate tumor burden. Deep-red FPs are more suitable for *in vivo* applications as compared to eGFP and dsRed2.

## Introduction

Xenografts of human tumors in mice are important preclinical *in vivo* test models for the evaluation of new chemotherapeutic drugs and drug delivery systems. Subcutaneous (s. c.) xenograft tumor models are well described, easy to handle and suitable for a lot of applications. Xenograft tumor growth and response to therapy can be directly followed by observation. The tumor burden is evaluated by volume calculation using caliper measurement. Orthotopic xenograft tumor models, which are more difficult to establish, are close to the clinical behavior of malignant tumors in terms of location of the primary tumor site, the tumor progression, the tumor micro-environment, cell migration and metastasis [1]–[4]. Because metastatic spread is the main problem in tumor therapy, models are required where these aspects are considered. However, monitoring of xenograft tumor growth and evaluation of the whole tumor burden during an experiment is very difficult in such models. In order to utilize these models, sensitive, robust and quantifiable non-invasive monitoring methods are imperative requirements [5]–[7].

Non-invasive multispectral fluorescence imaging (MSFI) using genetically encoded fluorescence proteins (FP) is a promising tool to detect and monitor primary tumor growth as well as metastatic disease and their therapy response *in vivo*. Even simultaneous imaging of separated fluorophores reporting different model parts or processes, e. g. therapeutic agent, drug carrier system and therapy target, is possible [8]–[10]. Previously published fluorescence imaging data of orthotopic tumor models using genetically encoded FP often focus on the chances and limits of primary tumor and metastasis detection [11]–[15]. The spectral characteristics of the FP are the crucial limitation for successful *in vivo* monitoring by fluorescence imaging, and deep-red or near-infrared (NIR) light emitting FPs offer the best spectral features because they relate to the so called optical window for fluorescence imaging of deeper tissues [16]–[21]. Further studies dealt with the correlation of different imaging methods and compared their advantages and disadvantages [5], [22], [23].

However, there are only few reports on the non-invasive *in vivo* monitoring of tumor growth over the complete study period which provide a relevant quantification of the data, i.e. evaluation of tumor volume/burden based on measured imaging signals [23]–[25]. Moreover, detailed investigations to establish specific correlations between intrinsic fluorescence signals and tumor burden with the aim to eventually monitor tumor growth and therapy response only by imaging are not reported. Such an application requires that the fluorescence signal produced by a FP expressing tumor corresponds to the respective tumor volume independent of different phases of tumor growth. The aim of the present study was to investigate whether the kinetics of tumor growth can be followed non-invasively by *in vivo* fluorescence imaging and which requirements need to be fulfilled. A prerequisite was to demonstrate that our reporter and imaging system employing stable ubiquitin promoter driven cellular FP expression provides robust and quantifiable imaging data *in vivo*. For this purpose we used the s. c. xenograft tumor model since it allows two independent monitoring methods in parallel: First, the calculated tumor volume based on caliper measurement as decisive tumor burden parameter and second, the fluorescence intensity (FI) measured by non-invasive *in vivo* imaging. Different cell lines as well as four FPs differing in excitation/emission spectra (eGFP, dsRed2 and the deep-red FP TurboFP635 and mPlum) were tested. Furthermore, a model comprising multiple s. c. tumors in an individual animal was used since metastatic disease would result in various progressive, fluorescent sites. As an easy-to-handle model for tumors growing inside the body, the cell suspensions were injected intraperitoneally (i. p.) to generate visceral growing tumors. In addition to tumor progression the chemotherapeutic response was examined in the s. c. and i. p. xenograft models. Finally, possible sources of error were analyzed and statistically tested.

## Materials and Methods

### Cell Culture and Fluorescence Protein Expression

The following human tumor cell lines were used: DLD-1, HT-29 (both colorectal carcinoma, CRC; ATCC CCL-221 and ATCC HTB-38 (http://www.lgcstandards-atcc.org/); re-authenticated by the DSMZ (http://www.dsmz.de/services) using STR analysis in 2010) and H12.1 (nonseminomatous germ cell tumor, GCT; [26]). Cell lines were maintained as monolayer cultures in RPMI 1640 (PAA, Pasching, Austria) supplemented with 10% fetal bovine serum (Biochrom AG, Berlin, Germany) and 1% streptomycin/penicillin (PAA, Pasching, Austria). Cultures were grown at 37°C in a humidified atmosphere of 5% CO_2_/95% air. Cells were stably marked with FPs (eGFP, dsRed2, TurboFP635 or mPlum) by lentiviral transduction using the vector system and the method as described previously for eGFP and dsRed2 [27]. The cDNAs of the deep-red FPs TurboFP635 and mPlum were obtained from Evrogen (Evrogen Joint Stock Company, Moscow, Russia) and Clontech (Takara Bio Europe/Clontech, Saint-Germain-en-Laye, France) respectively, and cloned into the vector system. After transduction the respective strong fluorescent cell clones were selected, characterized *in vitro* and then used for the *in vivo* experiments. The *in vitro* characterization included cytotoxicity assays to show that cell line specific sensitivities to chemotherapeutic agents are retained. For example, the H12.1 eGFP cells retained their hypersensitivity to cisplatin, which is a hallmark of germ cell tumor cells. In contrast, HT-29 eGFP colorectal carcinoma cells are more resistant to cisplatin (5-fold difference in apoptosis inducing IC90 dose).

### Animal Handling

All experiments were performed according to institutional guidelines. The study had been approved by the animal care and use committee of Saxony-Anhalt, Germany (Permit Numbers: AZ 42502-2-773 MLU & AZ 42502-2-920 MLU). Xenograft tumors were established in male athymic Nude-Foxn1 mice (Harlan Winkelmann, Borchen, Germany) by s. c. (5*10^6^ tumor cells in 100 µL PBS) or i. p. (1*10^6^ tumor cells in 100 µL PBS) injections. Monitoring of tumor growth was performed at least twice per week. The growth of the s. c. tumors was followed by means of caliper measurements and volume calculation (V = w^2^ * l * π/6 with w as tumor width and l as tumor length) as well as fluorescence imaging at the same time point. The i. p. growing tumors were monitored only by fluorescence imaging.

Cisplatin therapy was administered i. p. as a single dose of 10 mg/kg body weight. The combination chemotherapy regime comprised an i. p. injection of Irinotecan (50 mg/kg body weight) and 5-Fluorouracil (30 mg/kg body weight) on day 1 followed by 5-Fluorouracil single treatment on day 2, 3 and 4. All three drugs were obtained from Sigma-Aldrich Chemie (Taufkirchen, Germany).

Body weight and the general condition of mice were assessed at least twice a week except for animals being under therapy, which were monitored daily. Finally, all mice were sacrificed and underwent necropsy. Tumors were removed and weighed, fixed in 5% buffered formalin and embedded in paraffin for further analyses.

### Fluorescence Imaging

Non-invasive *in vivo* multispectral fluorescence imaging was performed with a Maestro™ *in vivo* fluorescence imaging system (CRi, Inc., Woburn, USA) using Maestro™ software version 2p22 or the updates 2.4.3 and 2.10.0, respectively. The specific feature of the Maestro™ imager is that a collection of spectral data at different wavelengths, called cube, is taken rather than a single image at a determined excitation and emission wavelength. This enables the isolation of various fluorescence signals and therefore better signal-to-noise ratios can be achieved, e. g. through autofluorescence removal [10].

Mice were anesthetized for the imaging procedure either with an i. p. injection of Ketamine (75 mg/kg body weight; Bayer Animal Health GmbH, Leverkusen, Germany) and Xylazine (5 mg/kg bodyweight; Pfizer Deutschland GmbH, Berlin, Germany) or with a vapor Isoflurane inhalation narcosis system (Drägerwerk AG, Lübeck, Germany). Initially, 2.5% Isoflurane (Abbott GmbH & Co. KG, Wiesbaden, Germany) in O_2_ with 2 to 3 L/min were given via inhalation mask coupled on a black warming plate (35°C). For narcosis preservation the Isoflurane level was reduced to 0.8 to 2% depending on the individual animal and the narcosis depth and duration. For eGFP imaging the blue filter set (bl) was used with the extinction band pass filter 435 to 480 nm, the emission filter 490 nm longpass and the acquisition settings 500 to 720 nm in 10 nm steps. For dsRed2 imaging the green filter set (gr) was used with the extinction band pass filter 503 to 548 nm, the emission filter 560 nm longpass and the acquisition settings 560 to 750 nm in 10 nm steps. The yellow filter set (ye) was used for TurboFP635 and mPlum with the extinction band pass filter 575 to 605 nm, the emission filter 645 nm longpass and the acquisition settings 630 to 850 nm in 10 nm steps. Instrument settings were specified as: specimen stage 1C, position of lamps 2 and binning 2×2. Each sedated mouse was focused and a grayscale image was taken with 10 or 30 ms exposure time under white light. Afterwards, the white light was changed to excitation light. The exposure time [ms] was measured automatically. Cubes were taken with the highest integer ms without over exposure.

### Imaging Data Analysis

All cube analyses were performed with the Maestro™ software mentioned above. The discrete fluorescence signals were isolated from the cubes using filter specific spectral libraries and the unmix function of the software. The libraries contain (a) murine autofluorescence, (b) black warming plate (corresponds with background/baseline) and (c) the pure spectra of the fluorophore to be measured, which was also recorded *in vitro* prior to the measurement. These signals result in 12-bit grayscale images (696×520 pixel) for each spectrum which were analyzed and quantified. First, an unmixed composed image was created by allocation of colors to each signal: autofluorescence - white, background - black, eGFP - green, dsRed2 - red, TurboFP635 - light magenta and mPlum - deep magenta. These unmixed composed images clearly visualized the localization of the measured fluorophore. For quantification the Maestro™ software measure tool was used. The total fluorescence intensity (FI) of the whole image given as summation of all single pixel intensities was normalized on exposure time ([ms^−1^]) and saved for further analyses. No specific region of interest (ROI) selection was performed as a result of the two following considerations. Foremost, the target xenograft models with metastatic disease will result in multiple fluorescent sites presumably with varying intensities due to their different localizations in the mouse body. The latter are not predictable and therefore the usage of a certain threshold level is not feasible. Moreover, the decision for a true signal should be objective, independent of the investigator and therefore a manually placed ROI is not preferable. In addition, the overlay images of grayscale pictures under white light and isolated fluorescence signals in pseudocolor jet black mode were generated with GNU Image Manipulation Program (GIMP, version 2.6) for better comparable pictures with color scaled fluorescence intensities. Image conversions, rotations, scaling etc. were carried out with ImageMagick (version 6.7.4).

### Procedures for Error Analyses

All descriptive analyses describe mean [ms^−1^] and standard deviation (S. D. [ms^−1^]) of the FI data generated by a five times repeated individual mouse imaging at the one time point. Mixed models with fixed and random effects were fitted to describe repeated logarithmized measurements of FI parameters [28]. Measurements were repeated with various s. c. tumors in different mouse individuals. A common correlation among the observation from a single mouse was assumed corresponding to the compound-symmetry structure. Due to the non-convergence of one mixed model two mixed models were calculated. To describe the influence of different fluorescence colors, cell lines and tumor volumes on the FI error (S. D. [%]) ANOVA f-type statistics were used. Calculations were made with Predictive Analysis Software 18 (PASW 18, 2009 version of SPSS, IBM).

**Figure 1:**
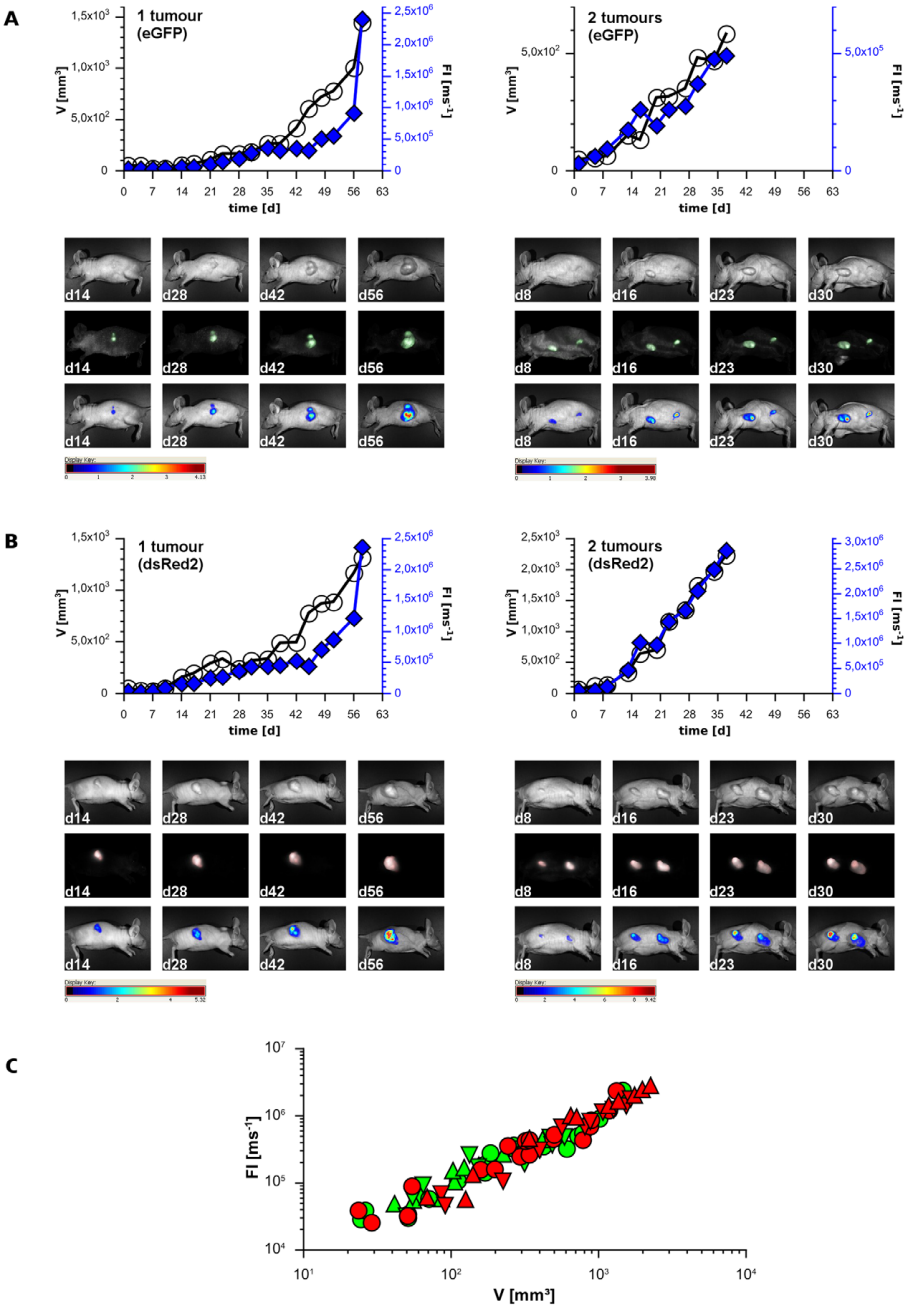
Monitoring of tumor growth by fluorescence imaging in the s. c. DLD-1 xenograft model. Representative data sets of one and two tumors expressing eGFP (A) or dsRed2 (B) are displayed. The calculated tumor volume V (○) and the measured total FI normalized on exposure time (♦) were plotted over time. For demonstration of the method exemplary grayscale images (exposure time 10 ms), unmixed composite images (automatic exposure time; background - black, autofluorescence - white, eGFP - green, dsRed2 - red) and overlay images (intensity weighted pseudocolor mode, scale bar displayed beneath) of one mouse at various time points are given under each graph. In further presentations only representative pseudocolor images, which are most illustrative, will be displayed. As shown in A and B, green and red fluorescence signals increased over time like the tumor volumes. (C): Correlation between FI and V (10 tumors in 3 individuals representing two single tumor sites (circles) and four combined tumor sites (up and down triangles), eGFP FI - green symbols, dsRed2 FI - red symbols).

## Results

### Preliminary Considerations: A Simple Quantification Model of Stably Fluorescent, Growing Xenograft Tumors

Two simplified assumptions were considered: (A) An optimal growing tumor has an exponentially increasing volume V over time. (B) Since each tumor cell is stably marked with a genetically encoded FP under a constitutive promoter, the FI of the whole tumor grows exponentially over time as well. V and FI are therefore functions of time. Finally, rearrangement of equations leads to a power function for the FI as function of V with b being the exponent, which is defined by the ratio of the specific growth rates µ_FI_ and µ_V_, respectively (equations 1c). Linearity occurs only if both values increase equally fast, i.e. b = 1.

**Table 1:** Data of potential fits for all analyzed HT-29 s. c. xenografts.

FP	mouse	b	R^2^	V_max_ [mm^3^]	intracutaneous tumor parts
eGFP	1_1_	1.43	0.89	244	++
	2_1_	1.39	0.94	570	++
	3_1_	1.07	0.88	192	+++
	4_1_	1.05	0.93	243	−
	5_1_	0.77	0.90	442	+
	6_1_	0.77	0.82	359	−
	7_1_	0.71	0.91	1204	−
	8_1_	0.51	0.86	282	−
	9_1_	0.44	0.75	1014	−
	10_1_	0.42	0.52	282	−
	all	0.76	0.61	1204	−to +++
dsRed2	1_1_	1.31	0.64	103	+
	2_1_	0.67	0.42	110	−
	3_1_	0.67	0.74	180	−
	1_3_	1.96	0.89	∑600	−
	2_3_	1.73	0.97	∑424	−
	3_3_	1.18	0.92	∑883	−
	all	0.80	0.73	∑883	− to +
TurboFP635	1_1_	1.07	0.93	1622	−
	2_1_	0.79	0.93	1055	−
	3_1_	1.14	0.94	1281	−
	all	1.01	0.88	1622	−
mPlum	1_1_	0.63	0.86	693	−
	2_1_	0.80	0.93	244	−
	3_1_	0.63	0.86	402	−
	all	0.77	0.87	693	−

The individual development of s. c. tumors in different mice is clearly shown: Final V as well as the exponents b of the function f (V) = FI varied markedly. FP: fluorescence protein; mouse: individual with indices 1 or 3 for number of tumor sites; b: exponent of the potential function f (V) = FI = a * V^b^ with b = µ_FI_/µ_V_; R^2^: coefficient of determination (potential regression by transformation into a linear model using LN, R^2^ (LN(y);LN(x)); V_max_: maximum volume in mm^3^ (∑: all three calculated V were summed up); intracutaneous tumor parts: classification of intracutaneous, highly fluorescent tumor parts (-: 0% of tumor surface, +: <<50% of tumor surface, ++: <50% of tumor surface, +++: approx. 50% tumor surface).

**Figure 2:**
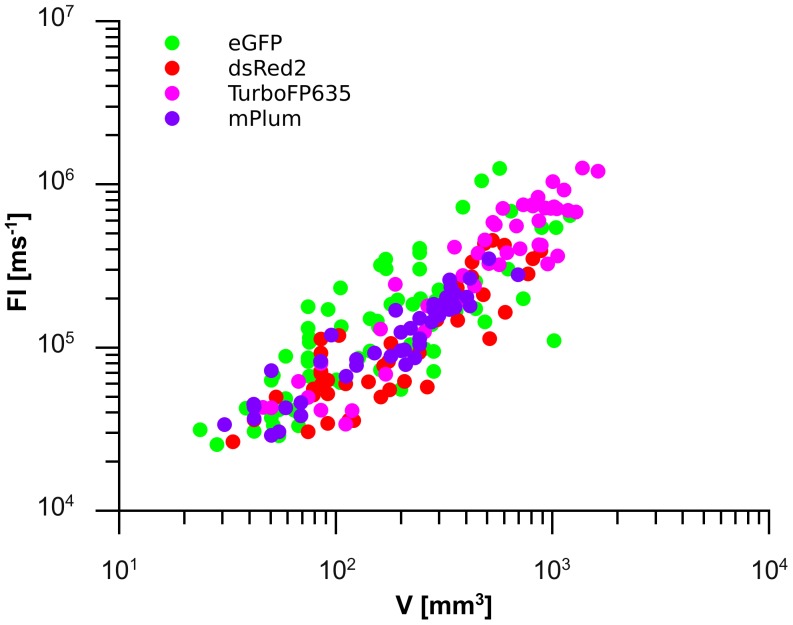
Fluorescence color dependency of the correlation between V and FI for the HT-29 s. c. xenograft model. Data are plotted for eGFP, dsRed2, TurboFP635 and mPlum emitting tumors with disregard of mouse individuals. It is obvious that the scattering of deep-red data points is smaller than those of the green and red fluorescent xenografts, respectively.

The resulting potential approach was investigated in s. c. xenograft models where V and FI were monitored in parallel.
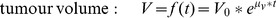
(1a)

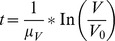


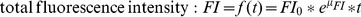
(1b)


ansatz total fluorescence intensity as function of tumour volume:
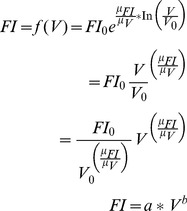
(1c)


### Following Xenograft Tumor Growth by Fluorescence Imaging–proof of Principle

A group of three mice were employed to validate our setting using pure fluorescence signals for tumor quantification with taking total FI normalized on exposure time as the summation of all pixel signals. One mouse had two s. c. DLD-1 xenograft tumors: one expressing eGFP on the left side and one expressing dsRed2 on the right side (Fig. 1A and B, each left). Two mice had four s. c. DLD-1 xenograft tumors: two expressing eGFP on the left side and two expressing dsRed2 on the right side (Fig. 1A and B, each right). The growth of xenografts was followed by caliper measurement and volume calculation. In case of two tumor sites on the same flank each tumor was measured separately and then volumes were added to get whole tumor burden. This was allocated to the respective fluorescence signal, which always was taken as total FI. As shown in Figure 1 both values V and FI increased equally fast and correlated well over the observation time. This finding was independent of the type of FP as well as the number of tumor sites. Finally, all FI values obtained from single and combined tumors were plotted over their respective V values. This demonstrated the correlation of both data sets (Fig. 1C). Hence, analyzing eGFP FI as well as dsRed2 FI can be used to substitute V for monitoring tumor growth in the s. c. DLD-1 xenograft model.

**Figure 3:**
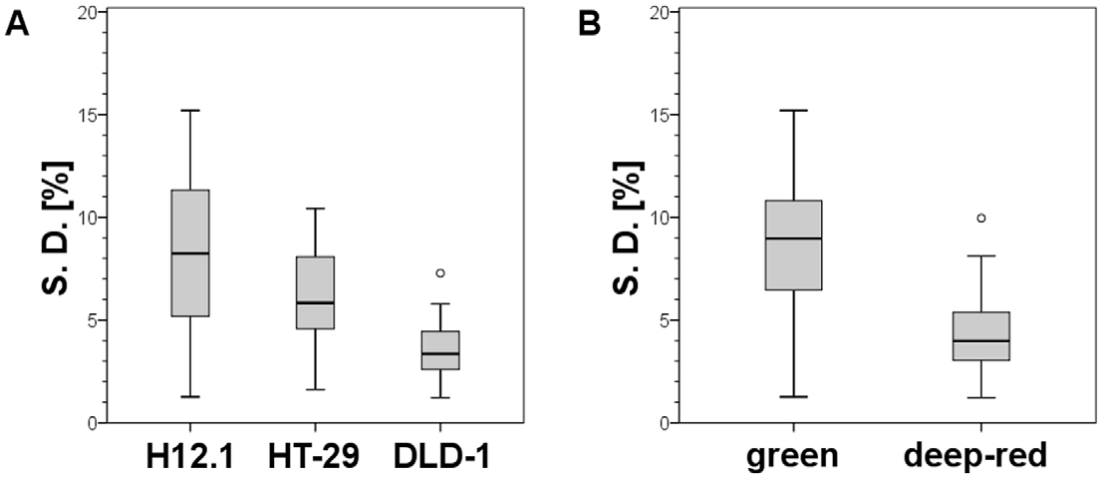
Standard deviations of the FI as a function of the cell type and the fluorescent color. The scattering of the S. D. of 5 different cubes based on the mean FI is shown. Thick black line: median of S. D. [%] (50th percentile), box bottom: 25th percentile, box top: 75th percentile, whiskers: 1.5× interquartile range, circle: outliers. (A): A great influence of the chosen xenograft model on the imaging error is observed. Each model has its specific growth characteristics (e. g. growth rate, vascularization or recruitment of murine stroma) and therefore the optical properties of the developing solid s. c. tumor differ greatly resulting in the observed variation. (B): Different fluorescence colors have specific optical properties in living tissues (e. g. penetration depth of excitation light or absorption of emitted fluorescence). This can lead to color dependent varying FI errors as shown here for green and deep-red fluorescence demonstrating superior characteristics of the latter.

### Correlation of FI and V–influence of the Tumor Cell Model and the Type of Fluorescence

To further investigate the correlation between FI and V the next study was performed using a larger series of individuals and another cell line model. A group of 14 mice were inoculated s. c. with HT-29 eGFP cells to generate single tumors. In two mice no tumor growth was observed during the monitoring period (6 weeks). Additionally, two mice had fast intracutaneous growing tumor parts leading to over exposure even with the shortest exposure time possible (1 ms). Therefore, images of these individuals could not be unmixed and analyzed. For the remaining 10 individuals FI was plotted over V and a potential function was fitted for each individual (Table 1). A good individual-specific correlation of FI and V was observed in 8 out of 10 mice as determined by a R^2^ coefficient ≥0.80 (Table 1). However, an obvious inter-individual variation exists which is indicated by different exponents b of the function f (V) = FI and different final tumor volumes (Table 1). Therefore, in contrast to the study with DLD-1 tumors (see Fig. 1C) a combined analysis of all data sets from HT-29 eGFP tumors leads to a poor correlation with a total R^2^ = 0.61 (Table 1) and as illustrated in Figure 2 by the higher scattering of green symbols.

**Figure 4:**
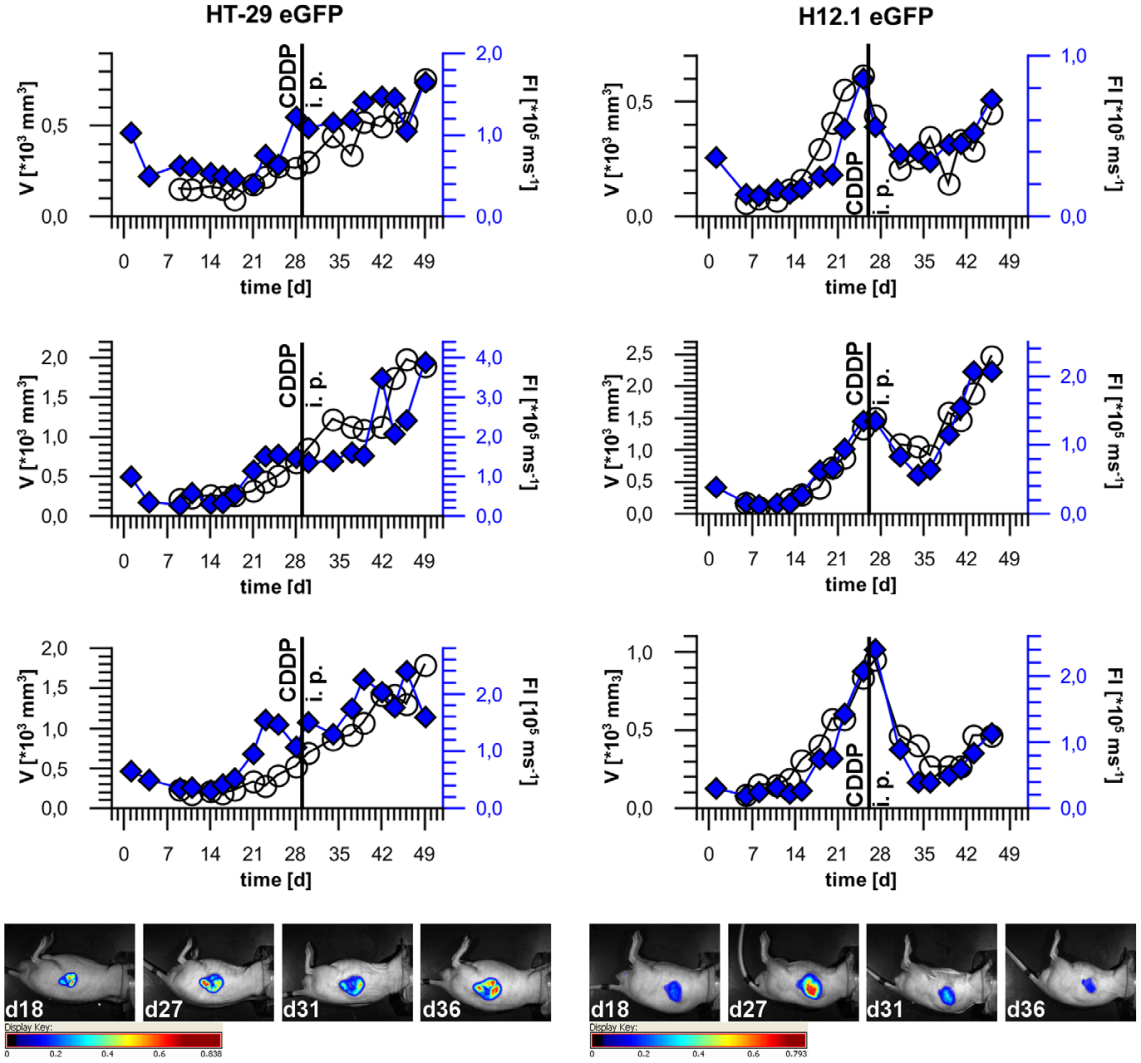
Monitoring of s. c. HT-29 eGFP and H12.1 eGFP xenografts after chemotherapy with Cisplatin (10 mg/kg body weight) *in vivo*. The calculated tumor volume V (○) and the measured normalized total fluorescence intensity FI (♦) were plotted over time. Each plot represents one treated individual. Day of chemotherapy is marked with a vertical line (d29 or d26 post cell implantation, respectively). In the last row representative intensity weighted overlay images are shown corresponding to the plots in the row above.

In addition, three mice, each with four s. c. HT-29 dsRed2 tumors (one single tumor on the right flank and three tumors on the left flank, respectively) were analyzed. For the three tumors on the same flank V was given as summation of single tumor V whereas the fluorescence signal was taken as total FI. Similar to the study with HT-29 eGFP xenografts an obvious inter-individual variation was observed with HT-29 dsRed2 cells derived tumors (Fig. 2, red symbols; Table 1) leading to poor, even though somewhat improved, correlation when all data sets were combined (total R^2^ = 0.73). Interestingly, total FI derived from three tumors correlated better with V than signals from a single tumor. An obvious reason for that are the very small V for the single tumors in the three analyzed individuals and the resultant large errors in calculation of V (see below: Evaluation of potential measurement errors).

Furthermore, the correlation of FI and V was investigated in a third tumor cell model comprising s. c. eGFP expressing H12.1 xenografts. As in the HT-29 model an obvious inter-individual variation was observed for the H12.1 model with a total R^2^ = 0.72 (n = 6).

**Figure 5:**
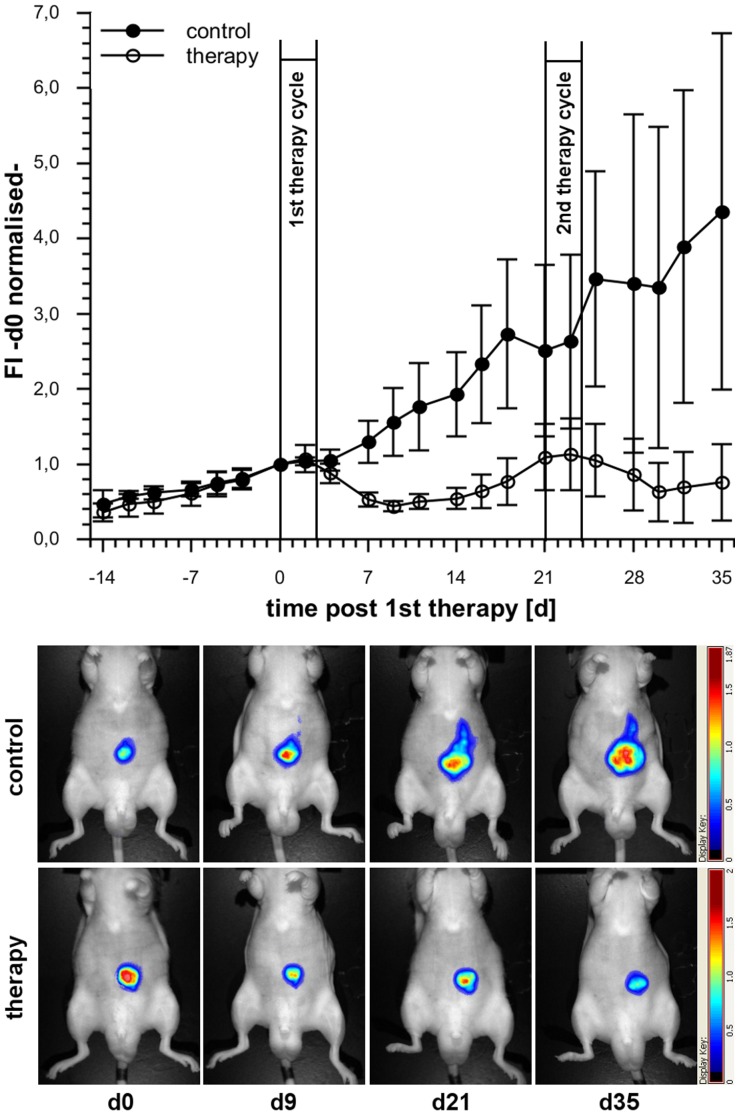
Monitoring of growth and chemotherapy response in the model of visceral growing mPlum HT-29 xenografts. Combination therapy was given i. p. in two cycles (d0 - d3 & d21 - d24): On the first day the animals received an i. p. injection of Irinotecan (50 mg/kg body weight) and 5-Fluorouracil (30 mg/kg body weight) followed by 5-Fluorouracil single agent applications on day 2, 3 and 4. The plot shows the development of the normalized FI for the therapy (○; n = 7) and the control group (•; n = 4). Error bars are given as S. D. The overlay images show one representative individual per group. It becomes apparent that chemotherapy induced a clear decrease of FI indicating the response of tumors. Compared to untreated mice a successful prevention of tumor growth was achieved by the chemotherapy regimen.

**Figure 6:**
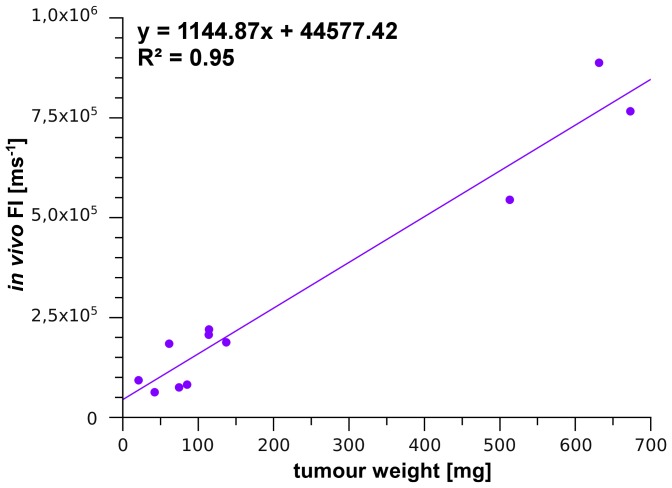
Correlation between FI and tumor weight. The FI were plotted over the weights of the tumors that had been detected *in vivo* based on their mPlum signal (n = 11).

The next study focused on deep-red FPs since those are thought to be more suitable for *in vivo* studies due to a better signal penetration. We were able to generate cell clones from the cell lines DLD-1 and HT-29 expressing the deep-red FPs TurboFP635 and mPlum. According to the procedures described above, the correlation of FI and V were investigated in the respective s. c. xenograft model. Similar to the studies with eGFP and dsRed2, for the DLD-1 TurboFP635 and mPlum xenograft models a good correlation of FI and V was observed (total R^2^ = 0.89 and total R^2^ = 0.89, respectively). Interestingly, in the HT-29 model the application of TurboFP635 and mPlum expressing xenografts resulted in an improved correlation of FI and V (total R^2^ = 0.88 and 0.87, resp.) and led to a reduced inter-individual variation as demonstrated in Figure 2 (magenta and violet symbols) and Table 1.

### Evaluation of Potential Measurement Errors

Assuming exponential tumor growth over time and a constant fluorescence intensity of each single tumor cell the correct simplified correlation model between V and FI is a potential ansatz (see equations 1). The error dimensions of both values and their dependencies have to be considered in order to judge the quality of function fitting and parameter calculation.

The V deviation can be determined using error propagation with consideration of the investigator dependent handling of tumor measurement. The volume error [%] results in a sum of twice width (w) and single length (l) error percentage (Δ =  ±0.5 mm). This leads to greater errors in small V compared to large ones (see equations 2b).
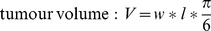
(2a)

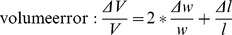
(2b)


The FI error can depend on the individual mouse and may be influenced by the exact tumor position, occurrence of hemorrhage or shadows caused by optical dense tissues such as bones. Additionally, the mouse positioning in the imager, the tumor volume and its self-light absorption, the xenograft model (cell clone), the spectra isolation algorithm as well as the fluorescence color may also influence imaging reliability. To evaluate the FI error a black-box experiment was performed (see Materials and Methods, Procedures for error analyses) where 315 cubes were generated resulting in 63 mean FI and the corresponding S. D. for 23 mouse individuals partly measured at different time points.

Initially, the relation between FI and V, xenograft model and fluorescence color was investigated. A statistically significant influence of the xenograft model (p = 0.006), the tumor volume (p<0.001) but no influence of the fluorescence color (p = 0.671) on FI was shown. In the second one a strong FI dependency was found for V and the xenograft model (both p<0.001). These significant results affirm the observed individually correlated increase of V and FI depending on the chosen xenograft model. (see Fig. 1C, 2 and Table 1).

Next, an UNIANOVA analysis of the ascertained S. D. [%] was performed, which indicated a significant influence of the xenograft model (p = 0.032) and the fluorescence color (p = 0.003). The influence of the chosen xenograft model is shown in Fig. 3A. Figure 3B demonstrates the superior characteristics of deep-red fluorescent tumors with smaller S. D. [%] mean and scattering of those compared to green fluorescent tumors. No significant influence of V on the S. D. [%] was observed (p = 0.527; data not shown). The tendency of decreasing S. D. [%] with increasing FI was not significant (p = 0.264; data not shown).

Finally, weekly repeated error imaging of one and the same animal with a growing s. c. tumor showed varying S. D. [%], but a mouse specific error was not noticed neither for any fluorescence color nor for any xenograft model (data not shown).

In conclusion these analyses show a clear cell model dependent probability for FI measurement errors and suggest that these variations may contribute to cell model specific inter-individual variations of FI/V relations leading to poor correlations of FI and V when all data of a series were combined. In addition, the combined data demonstrate that both the inter-individual variation of FI/V relations and the probability for FI measurement errors is diminished when using deep-red fluorescent tumors.

### Monitoring of Chemotherapy Response by Non-invasive *in vivo* Fluorescence Imaging

In addition to monitoring of tumor growth, the analysis of response to therapy is another important aspect when using preclinical tumor models. Whether alterations in xenograft tumor growth in response to chemotherapy can be followed by analyzing FI was tested in the s. c. tumor models of highly cisplatin sensitive H12.1 eGFP cells and more resistant HT-29 eGFP cells. As shown in Figure 4 a single treatment with cisplatin induced transient growth retardations in HT-29 tumors and dramatic regressions in H12.1 tumors which was associated with retardations in FI increase and a clear FI decrease, respectively. Interestingly, a correlation of FI and V was also observed in case of tumor re-growth after regression (Fig. 4, right). These data show that analyzing FI can be used to substitute V for monitoring xenograft tumor growth and response to chemotherapy in s. c. tumors.

Based on these data the next study was performed to investigate whether growth and response to chemotherapy of tumors growing inside the body can be monitored by analyzing FI using deep-red fluorescent tumors. For this purpose HT-29 mPlum cells were injected i. p. to generate visceral growing tumors. A combination treatment of Irinotecan and 5-Fluorouracil was chosen as a clinical relevant chemotherapy regimen. As shown in Figure 5 the FI of the control group increased continuously over time whereas a clear decrease of FI was induced in response to chemotherapy. After re-increase of FI indicating re-growth of tumors a second course of chemotherapy again induced a decrease of FI (Fig. 5). Surprisingly, in some cases final necropsy revealed the existence of additional, deeper located tumors that had not been detected *in vivo* by their mPlum signal although they proved to be strong fluorescent *ex vivo*. Consistently, not the whole tumor burden but only the weights of the tumors that were detected *in vivo* based on their mPlum signal correlated with FI (Fig. 6).

In conclusion, as long as tumors are visualized by the fluorescence signal the FI can be used to follow growth and response to therapy.

## Discussion

The results of these studies demonstrate the applicability of MSFI for tumor monitoring under different experimental designs. First of all, we tested the proposed power function of our preliminary considerations to interrelate FI and V using total FI normalized by exposure time in s. c. growing tumor xenografts. Although this was only a theoretical approach, the measurement of the FI correlated well with the calculated V in the DLD-1 model. In addition, the non-invasiveness of the MSFI technique allowed numerous measurements over long monitoring periods without negative effects on the individual animal. Next, we investigated a possible influence of the chosen tumor cell model and fluorescence type. As our data clearly show, the imaging characteristic of a certain xenograft model and its quantification quality depends strongly on both parameters. Therefore, a generalization of the method is difficult and a specific FI/V interrelation has to be established for each distinct xenograft model, strictly speaking for each generated fluorescent cell clone. In the HT-29 model for example, the partly observed differences of the individual exponents decreased the correlation between both parameters. In order to judge the calculated correlations, the errors of V and FI have to be taken into consideration. The tumor caliper measurement may depend on the experimenter. The following V calculation has a relatively large error especially for small tumors. This has to be considered when determining the power function f(V) = FI. Moreover, the specific growth characteristics play a key role. As observed in the HT-29 model, in case of local invasive growth into the skin, the fluorescence intensity increases enormously. This can lead to overexposure and therefore, a correct signal isolation using fluorescence spectra is impossible. However, this only applies for s. c. models and can be neglected for tumors growing inside the body.

Obviously, the substantive simplification of our preliminary considerations has limits which may have contributed to differences in FI/V correlations depending on the model. First, the single cells within the tumor may differ in their FI. Furthermore, a xenograft consists not only of fluorescent tumor cells but recruits e. g. blood vessels and non-fluorescent murine stroma. Necrotic/fibrotic areas can develop during tumor progression which becomes non-fluorescent as well. Moreover, all these processes are not stable so that structural rearrangements occur in tumors over the monitoring period.

However, all cell model specific negative influences appear to be reduced when applying deep-red fluorescent cells. This was also reflected by the results of a performed error analysis. A possible explanation for this phenomenon could be that in deep-red tumors more cells contribute to the total fluorescence signal since also deeper located cells are detected making the measured total FI more robust and less dependent of structural rearrangements. Thus, deep-red FPs have superior characteristics for *in vivo* studies for two reasons: the better tissue penetration of excitation light and emitted fluorescence signal due to longer wavelengths [29], [30] and in diminishing cell model specific effects.

Finally, we investigated a model of visceral growing HT-29 mPlum tumors. The results show that the MSFI provided parameter FI can be used for monitoring of xenografts growing inside the body, even under therapy. The tumor weights of detected tumors correlated with FI. However, in some cases final necropsy revealed the existence of additional, deeper located tumors that were not visualized before. These deep located tumors showed a strong deep-red fluorescence *ex vivo. In vivo* this fluorescence was masked by the intestinal tract. Thus deep-red FPs, at least as used in our models, are not always sufficient to detect all tumor sites in the murine body. This led us to the final conclusion that MSFI can be used for tumor monitoring as long as a true fluorescence signal can be isolated and its quantification is feasible.

Nevertheless, deep-red FPs are indeed more sensitive as the short wavelength FPs such as eGFP or dsRed2 as demonstrated previously [29], [30]. We were able to confirm this, performing mouse cadaver experiments comparing the four FPs described in our study (data not shown). For visualization of the deep located tumors in our model, an application of NIR based techniques might be an alternative method. In this regards, the new NIR emitting FPs IFP1.4 and iRFP as reported by Shu et al. [31] and Filonov et al. [16] respectively, are very promising tools. Therefore our next experiments will be performed using NIR fluorescent xenografts. Then a specific interrelation of FI and V has to be re-established with considering the specific features of NIR optics, especially the problem of 2D projection of a 3D fluorescence signal [17].
